# Oral Preexposure Prophylaxis Uptake and Discontinuation in the HIV Vaccine Trials Network 704/HIV Prevention Trials Network 085 Study: Implications for Biomedical Human Immunodeficiency Virus Prevention Trials

**DOI:** 10.1093/ofid/ofae387

**Published:** 2024-07-16

**Authors:** Valeria D Cantos, Moni Neradilek, Yunda Huang, Alison C Roxby, Kevin Gillespie, Allan C deCamp, Shelly T Karuna, Srilatha Edupuganti, Jorge Gallardo-Cartagena, Jorge Sanchez, Carlos del Rio, Valdilea Veloso, Myron S Cohen, Deborah J Donnell, Lawrence Corey, Colleen F Kelley

**Affiliations:** Division of Infectious Diseases, Emory University School of Medicine and Grady Health System, Atlanta, Georgia, USA; Fred Hutchinson Cancer Research Center, Seattle, Washington, USA; Fred Hutchinson Cancer Research Center, Seattle, Washington, USA; Fred Hutchinson Cancer Research Center, Seattle, Washington, USA; Division of Infectious Diseases, University of Washington, Seattle, Washington, USA; Fred Hutchinson Cancer Research Center, Seattle, Washington, USA; Fred Hutchinson Cancer Research Center, Seattle, Washington, USA; Fred Hutchinson Cancer Research Center, Seattle, Washington, USA; Division of Infectious Diseases, Emory University School of Medicine and Grady Health System, Atlanta, Georgia, USA; Centro de Investigaciones Tecnológicas Biomédicas y Medioambientales, Lima, Peru; Centro de Investigaciones Tecnológicas Biomédicas y Medioambientales, Lima, Peru; Division of Infectious Diseases, Emory University School of Medicine and Grady Health System, Atlanta, Georgia, USA; Instituto Nacional de Infectología Evandro Chagas, Fiocruz, Rio de Janeiro, Brazil; Division of Infectious Diseases, University of North Carolina, Chapel Hill, North Carolina, USA; Division of Infectious Diseases, Emory University School of Medicine and Grady Health System, Atlanta, Georgia, USA; Fred Hutchinson Cancer Research Center, Seattle, Washington, USA; Division of Infectious Diseases, Emory University School of Medicine and Grady Health System, Atlanta, Georgia, USA

**Keywords:** clinical trials, HIV, PrEP uptake, PrEP discontinuation, AMP

## Abstract

**Background:**

HIV Vaccine Trials Network (HVTN) 704/085, a placebo-controlled clinical trial assessing the efficacy of VRC01 broadly neutralizing antibody infusion for HIV prevention, offered oral preexposure prophylaxis (PrEP) as the standard of prevention at no cost to participants.

**Methods:**

We characterized features of- identified factors associated with- PrEP initiation and discontinuation, and the effects of PrEP initiation on HIV incidence.

**Results:**

Of 2221 participants, 31.8% initiated oral PrEP during study follow-up, with the highest proportion of PrEP initiations in Brazil (83.2%) and the United States (US) (54.2%). Prior PrEP use was associated with PrEP initiation (hazard ratio [HR], 2.22 [95% confidence interval {CI}, 1.25–3.95]). Participants from Switzerland (HR, 0.5 [95% CI, .3–1.0]) and Peru (HR, 0.08 [95% CI, .06–.1]) had lower likelihood of PrEP initiation compared to the US, while participants from Brazil had higher likelihood (HR, 2.6 [95% CI, 2.0–3.3]). In the US, PrEP initiation was lower in areas with higher unmet need for PrEP (HR, 0.9 per 5 units [95% CI, 0.8–1.0]). PrEP initiators had 58% less risk of acquiring HIV than PrEP noninitiators. Among PrEP initiators, 34.4% discontinued PrEP during study follow-up. Brazil had 63% less likelihood of PrEP discontinuation than the US (HR, 0.37 [95% CI, .22–.60]).

**Conclusions:**

When included as standard of prevention in HVTN 704/085, oral PrEP utilization patterns mirrored those observed in real-life settings. Variable effects of oral PrEP on HIV outcomes in clinical trials may be expected based on regional differences in oral PrEP use.

## Teaser

HVTN 704/085, a placebo-controlled HIV prevention study, offered oral preexposure prophylaxis (PrEP) as standard of prevention. Of all participants, 31.8% initiated PrEP and 34.5% of them discontinued PrEP during study participation. PrEP decreased the likelihood of HIV acquisition among PrEP initiators.

Oral [1, 2] and injectable [3, 4] preexposure prophylaxis (PrEP) is highly effective in preventing human immunodeficiency virus (HIV) acquisition; however, existing PrEP modalities may not fulfill the needs of all people due to comorbidities, limited local access, cost, or individual preference [4]. Therefore, additional innovative prevention strategies are needed to curb the global HIV epidemic. Anti-HIV-1 monoclonal broadly neutralizing antibodies (bnAbs) represent a promising alternative to antiretroviral-based prevention, especially for individuals looking for non-oral, longer-acting agents [5]. The Antibody Mediated Prevention (AMP) trials (HIV Vaccine Trials Network [HVTN] 704/HIV Prevention Trials Network [HPTN] 085 and HVTN 702/HPTN 081) consisted of 2 parallel phase 2b, multicenter, randomized, double-blind, placebo-controlled studies designed as proof-of concept studies to determine whether VRC01, a CD4 binding site–specific bnAb infused every 8 week over 20 months, was efficacious and safe to prevent HIV, when compared to placebo [6, 7]. Between 2016 and 2018, HVTN 704/HPTN 085 enrolled 2699 men who have sex with men (MSM) and transgender women (TGW) in Peru, Brazil, Switzerland, and the United States (US), while HVTN 703/HPTN 081 enrolled 1924 heterosexual cisgender women in sub-Saharan Africa [6]. Although VRC01 did not prevent HIV acquisition compared to placebo overall, it did provide 75% protection against VRC01-sensitive HIV viruses [6].

The establishment of emtricitabine/tenofovir disoproxil fumarate's (F/TDF) efficacy as oral PrEP changed the landscape of HIV prevention clinical trials design, as its inclusion in studies became an ethical obligation to minimize risks and maximize benefits to study participants [8–12]. The decision to include oral PrEP as an active comparator or to provide to all participants as part of the standard of care for HIV prevention depends on the experimental PrEP modality, availability of preliminary data indicating probability of protection, study phase and objectives, local regulatory status of different PrEP modalities, and study population [13]. Specifically, in studies assessing the safety and efficacy of experimental products for which no established effective intervention exists, such as preventive HIV vaccines and bnAbs, a placebo-controlled design is acceptable, and oral PrEP should be included as standard of prevention for all study arms. In contrast, in studies assessing the safety and efficacy of new antiretroviral PrEP modalities, an active comparator is preferred, as there are several antiretroviral PrEP products with established efficacy.

HVTN 704/HPTN 085 (henceforward: AMP) study offered oral PrEP with F/TDF to all participants as part of standard of care prevention [6]. During study design, regional and overall annual HIV incidence in the control group were estimated based on incidence data from prior HIV prevention clinical trials and accommodated various levels of PrEP uptake and efficacy. Overall, an assumption of 3% annual HIV incidence was used, which accommodated for up to 50% person-years at risk during which PrEP is used and PrEP efficacy of 90% during use [14]. Oral PrEP access plans for the study participants were developed by individual clinical trial sites and varied based on geographical region and year. In the US, oral PrEP access was established through an in-kind donation from Gilead Sciences linked to a mail-order pharmacy beginning at the study's onset. US clinical research sites had the option of directly providing PrEP services to study participants or referring to community PrEP clinics. In Switzerland, participants received PrEP through a nonprofit organization. In Brazil, oral PrEP was initially accessed through the PrEP Brasil Demonstration Project (ClinicalTrials.gov identifier NCT01989611) and in 2018, it became publicly available through the Brazilian Ministry of Health. In Peru, oral PrEP access was established through the PrEP Peru Demonstration Project in 2017 [6].

At the end of the study, HIV incidence was 2.35 per 100 person-years in the VRC01 group and 2.98 per 100 person-years in the placebo group, for an estimated prevention efficacy of 26.6%, which did not meet statistical significance [6]. Observed HIV incidence was similar to that predicted during study design; however, high geographic variability was seen, underscoring the need to better understand PrEP utilization patterns during the study. Overall PrEP uptake in the AMP study, measured by dried blood spot (DBS) levels of tenofovir-diphosphate in a subset of participants, was previously reported as 39% [6]. Significant regional differences in PrEP uptake were noted, with over 70% uptake in Brazil and less than 10% in Peru. Unlike DBS data, self-reported data on PrEP use were obtained prospectively for all enrolled participants, which allows for additional analysis to further characterize PrEP use among AMP trial participants. Here, we aimed to characterize PrEP initiation and discontinuation among AMP participants across geographic regions, to identify individual, clinical, and regional factors associated with PrEP initiation and discontinuation, and to determine the effects of PrEP initiation on HIV incidence and study retention.

## METHODS

### Study Overview and Design

We conducted a secondary data analysis of the AMP study. Sociodemographic, clinical, laboratory, site, and country/regional data were compared between enrolled participants who started PrEP at any point during their study participation up until the week 80 study visit (which corresponds to the study's primary analysis follow-up timepoint) and those who did not.

### Study Population

We included all AMP participants who received at least 1 study VRC01/placebo infusion. We excluded individuals who self-reported PrEP use during the enrollment visit or were not part of the primary analysis dataset. Additionally, participants who acquired HIV during the study or those diagnosed with HIV at the time of enrollment were excluded from the study retention analysis. Only participants who initiated PrEP were included in the PrEP discontinuation analysis.

### Patient Consent Statement

Consent was obtained from all AMP participants prior to the conduct of the study. This substudy does not include factors necessitating patient consent, as it is a secondary analysis of de-identified data.

### Outcome Measures

PrEP initiation was defined as the first medication start date for F/TDF for PrEP, starting at or after study enrollment, as reported by participants and entered into the PrEP/postexposure prophylaxis (PEP) electronic clinical research form (eCRF). For HIV incidence calculations, we used the primary study endpoint (documented HIV acquisition), which was previously confirmed by an independent adjudication committee, as described elsewhere [6, 14]. Study nonretention was defined as the date an individual stopped study participation prior to the completion of the study follow-up time (week 80 visit). Nonretention did not include those who stopped study participation due to inappropriate enrollment, incarceration, death, investigator decision, concerns about participants’ mental health, dual enrollment, or the coronavirus disease 2019 pandemic. PrEP discontinuation was defined as the first termination date of an in-study-initiated PrEP medication as entered in the PrEP eCRF [14].

### Covariates of Interest

The following covariates were considered as adjustments in some analyses: age in years, sex assigned at birth, gender identity, sexual orientation, race, ethnicity, treatment group (VRC01 vs placebo), region, behavioral risk score, and history of prior PrEP use. For US-based analyses, we also added the estimated PrEP-to-need ratio [15] for each clinical research site. The PrEP-to-need ratio is a measurement of the number of PrEP users divided by the number of people newly diagnosed with HIV in a certain year. It serves as an estimation of how PrEP use compares to the need for HIV prevention in a specific population or geographical area. Lower PrEP-to-need ratio denotes higher unmet need for PrEP [15]. Gender identity was self-reported along with sex assigned at birth. Gender categories included cisgender male, cisgender female, transgender male, transgender female, and additional self-reported identities. The baseline risk score, a proxy of an individual's likelihood for HIV-1 exposure, was calculated as defined previously [16]. Higher baseline risk scores indicate higher risk of HIV acquisition.

### Statistical Analysis

Baseline sociodemographic, clinical, and regional characteristics were summarized by median, range, and number (%) for continuous and categorical characteristics, respectively. Univariate and multivariate Cox proportional hazard models were conducted to identify factors associated with PrEP initiation and discontinuation. Predictors for both endpoints were selected using Cox least absolute shrinkage and selection operator (LASSO), with optimal lambda optimized deviance through 10-fold cross-validation. Separate models for PrEP initiation were fit: 1 for all participants, where we excluded the PrEP-to-need ratio as this variable is relevant to the US only, and a second model for US sites only, which included the PrEP-to-need ratio and excluded the region variable. To assess the effect of PrEP initiation on HIV incidence, we calculated HIV incidence rates before and after PrEP initiation. To quantify the effect of PrEP initiation, we used a Cox proportional hazard model of HIV incidence on time-varying indicator of PrEP initiation, adjusted for baseline behavioral risk score and study treatment allocation (VRC01 vs placebo). To evaluate the association between PrEP initiation and study retention, we calculated the total follow-up time for participants who initiated PrEP and those who did not. We also calculated incidence rates of study nonretention before and after PrEP initiation. We fit a time-varying Cox proportional hazard model, regressing study nonretention on time-varying PrEP initiation, adjusting for covariates with a *P* value <.1 in their univariate model.

## RESULTS

In a subset of 2221 enrolled participants who met the inclusion and exclusion criteria for this analysis, the median age was 27 years (range, 18–50 years), 99% were assigned male at birth, 89.4% identified as cisgender men, 74.4% were MSM, 26.4% were White, 14.8% were Black, and 63.3% identified as Latino. Of all participants, 42.8% of participants were enrolled at US sites, 49.2% in Peru, 6.4% in Brazil, and 1.5% in Switzerland (Table 1).

**Table 1.** Baseline Sociodemographic Characteristics Among Antibody Mediated Prevention Trial Participants Who Did Not Self-report Preexposure Prophylaxis (PrEP) Use at Enrollment, by PrEP Initiation

Characteristic	Noninitiators (n = 1515)	Initiators (n = 706)	All (n = 2221)
Age, y, median (range)	26 (18–50)	28 (18–50)	27 (18–50)
Assigned male sex at birth	1499 (98.9)	700 (99.2)	2199 (99.0)
Gender identity			
Cisgender male	1326 (87.5)	660 (93.5)	1986 (89.4)
Transgender male	9 (0.6)	3 (0.4)	12 (0.5)
Transgender female	144 (9.5)	19 (2.7)	163 (7.3)
Other/not assessed	36 (2.4)	24 (3.4)	60 (2.7)
Sexual orientation			
MSM	1093 (72.1)	560 (79.3)	1653 (74.4)
Bisexual	326 (21.5)	99 (14.0)	425 (19.1)
Straight	28 (1.8)	7 (1.0)	35 (1.6)
Other/not assessed	68 (4.5)	40 (5.7)	108 (4.9)
Race			
American Indian/Alaska Native	5 (0.3)	7 (1.0)	12 (0.5)
Asian	16 (1.1)	29 (4.1)	45 (2.0)
Black or African American	147 (9.7)	181 (25.6)	328 (14.8)
Multiracial	33 (2.2)	31 (4.4)	64 (2.9)
Native Hawaiian/Other Pacific Islander	3 (0.2)	3 (0.4)	6 (0.3)
Other	1040 (68.6)	140 (19.8)	1180 (53.1)
White	271 (17.9)	315 (44.6)	586 (26.4)
Hispanic or Latino	1142 (75.4)	264 (37.4)	1406 (63.3)
Region			
United States	435 (28.7)	516 (73.1)	951 (42.8)
Switzerland	21 (1.4)	13 (1.8)	34 (1.5)
Brazil	24 (1.6)	119 (16.9)	143 (6.4)
Peru	1035 (68.3)	58 (8.2)	1093 (49.2)
PrEP-to-need ratio^a^ (US only), median (range)	4.4 (0.8–24.8)	3.2 (0.8–24.8)	3.7 (0.8–24.8)
Baseline BRS^b^, median (range)	0.5 (−2.2 to 3.9)	−0.4 (−2.2 to 3.1)	0.2 (−2.2 to 3.9)
Treatment assignment			
Placebo	506 (33.4)	227 (32.2)	733 (33.0)
VRC01 (any dose)	1009 (66.6)	479 (67.8)	1488 (67.0)
Days to PrEP initiation, median (range)	NA	60 (1–673)	NA

Data are presented as No. (%) unless otherwise indicated.

Abbreviations: BRS, Behavioral Risk Score; NA, not applicable; MSM, men who have sex with men; PrEP, preexposure prophylaxis; US, United States.

^a^Lower PrEP-to-need ratio corresponds to higher unmet need for PrEP.

^b^Higher numbers indicate a higher likelihood of human immunodeficiency virus acquisition.

Of all participants, 31.8% (706/2221) initiated PrEP during their study participation (Table 1). Among PrEP initiators, median time to PrEP initiation was 60 days (range, 1–673 days) (Table 1).

Compared to noninitiators, PrEP initiators had a higher proportion of participants who were aged ≥25 years (73% vs 61%), who were cisgender men (93.5% vs 87.5%), who identified as MSM (79.3% vs 72.1%), who reported White race (44.6% vs17.9%), and who reported a history of PrEP use prior to enrollment (1.7% vs 0.2%). PrEP initiators had a lower median baseline sexual risk score. Among all PrEP initiators, 73.1% were enrolled at US sites, 16.9% in Brazil, 8.2% in Peru, and 1.8% in Switzerland (Table 1). Brazil had the highest proportion of PrEP initiations, with 83.2% (119/143) of all analyzed participants initiating PrEP during the study, whereas Peru had the lowest proportion of PrEP initiation (5.3% [58/1093]). Among US sites, those located in the southern US had the highest proportion of PrEP initiation (68.8% [207/301]), followed by those in the Northeast (51.6% [240/465]), West (42.4% [50/118]), and Midwest (28.4% [19/67]) (Supplementary Table 1).

**Figure 1.**
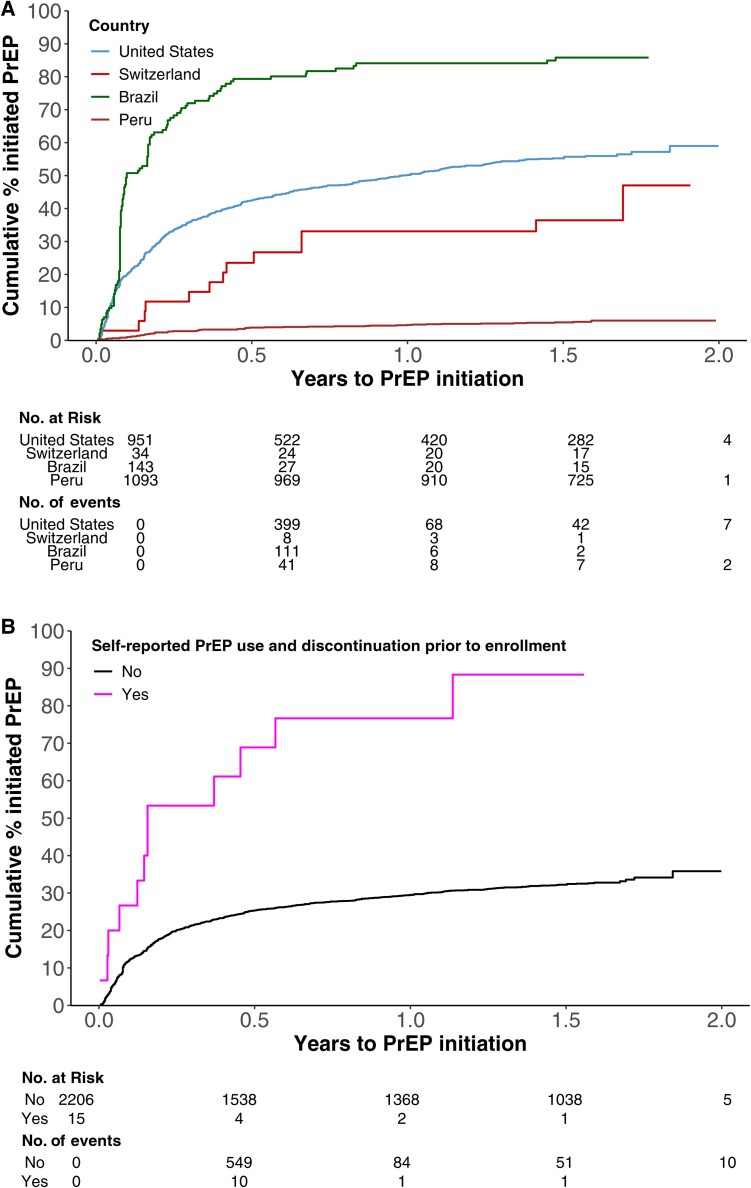
*A*, Preexposure prophylaxis (PrEP) initiation by country. *B*, PrEP initiation by prior PrEP use in the United States.

In the multivariate analysis conducted among all sites, region and history of prior PrEP use (hazard ratio {HR}, 2.22 [95% confidence interval {CI}, 1.25–3.95]) were associated with PrEP initiation. Compared to the US, Switzerland (HR, 0.55 [95% CI, .31–.97]) and Peru (HR, 0.08 [95% CI, .06–.12]) had a lower likelihood of PrEP initiation, while the Brazil sites had a higher likelihood of PrEP initiation (HR, 2.60 [95% CI, 2.01–3.35]) (Table 2 and Figure 1*A*). In the US, a history of prior PrEP use (HR, 1.95 [95% CI, 1.07–3.54]) (Figure 1*B*), and lower local PrEP-to-need ratio (HR, 0.90 [95% CI, .84–.97] per 5-unit increase), indicating a higher unmet need for PrEP, were associated with higher PrEP initiation (Supplementary Table 2).

**Table 2.** Multivariate Model for Preexposure Prophylaxis Initiation

Variable^a^	HR (95% CI)	*P* Value
Treatment assignment		
Placebo	1.00 (ref.)	
VRC01 (any dose)	1.04 (.88–1.21)	.671
Age, per 10 y	1.05 (.93–1.19)	.403
Male sex at birth	2.72 (.79–9.33)	.112
Gender identity		
Cisgender male	1.00 (ref.)	
Transgender male	1.35 (.26–7.15)	.722
Transgender female	0.74 (.45–1.22)	.236
Other/not assessed	1.30 (.84–2.03)	.238
Sexual orientation		
MSM	1.00 (ref.)	
Bisexual	0.87 (.70–1.08)	.221
Straight	1.17 (.53–2.61)	.699
Other/not assessed	0.82 (.58–1.16)	.267
Race		
White	1.00 (ref.)	
Black or African American	0.93 (.76–1.15)	.514
Other	0.86 (.67–1.10)	.239
Combined^b^	1.05 (.80–1.38)	.723
Country		
United States	1.00 (ref.)	
Switzerland	0.55 (.31–.97)	.038
Brazil	2.60 (2.01–3.35)	<.001
Peru	0.08 (.06–.12)	<.001
History of prior PrEP use	2.22 (1.25–3.95)	.007
Baseline BRS^c^, per 1 unit	0.98 (.85–1.14)	.802

Abbreviations: BRS, behavioral risk score; CI, confidence interval; HR, hazard ratio; MSM, men who have sex with men; PrEP, preexposure prophylaxis.

^a^Variables considered for least absolute shrinkage and selection operator selection: treatment, age, male sex at birth, gender identity, sexual orientation, race, Hispanic or Latino, region, history of prior PrEP use, baseline behavioral risk score.

^b^Combined: racial categories that accounted for less than 5% of the study population. These include American Indian/Alaska Native, Asian, multiracial, and Native Hawaiian/Other Pacific Islander.

^c^Higher numbers indicate a higher likelihood of human immunodeficiency virus acquisition.

The HIV incidence rate in the period pre-PrEP initiation was 3.6 per 100 person-years (PY) (95% CI, 2.9–4.5) and 1.2 per 100 PY (95% CI, .6–2.1) post-PrEP initiation (Supplementary Table 3). When adjusted for the baseline risk score and treatment assignment, participants who initiated PrEP had lower risk of acquiring HIV compared to noninitiators during study follow-up (HR, 0.42 [95% CI, .21–.82]; Table 3).

**Table 3.** Effect of Preexposure Prophylaxis Initiation on Human Immunodeficiency Virus Acquisition Risk

Variables	HR (95% CI)	*P* Value
PrEP initiation	0.42 (.21–.82)	.012
Baseline BRS^a^, per 1 unit	1.58 (1.26–1.98)	<.001
VRC01^b^	0.72 (.48–1.09)	.120

Abbreviations: BRS, behavioral risk score; CI, confidence interval; HR, hazard ratio; PrEP, preexposure prophylaxis.

^a^Higher numbers indicate a higher likelihood of human immunodeficiency virus acquisition.

^b^Treatment allocation (VRC01 at any dose vs placebo).

PrEP initiators had a median duration of study retention of 577 days (range, 28–801 days) compared to 562 days (range, 21–771 days) among noninitiators (Supplementary Table 4). In multivariate analysis, PrEP initiation had no effect on study retention (HR, 1.04 [95% CI, .78–1.39]; *P* = .77) (Supplementary Table 5). Among all participants who initiated PrEP during their study participation, 34.4% (243/706) discontinued PrEP at least once during the study period (Supplementary Table 1). By region, Brazil had the lowest proportion of participants who discontinued PrEP (18.5%) and Peru had the highest (58.6%) (Supplementary Table 1). In multivariate analysis conducted across all sites, region was associated with PrEP discontinuation. Compared to the US, Brazil had a 63% lower likelihood of PrEP discontinuation (HR, 0.37 [95% CI, .22–.60]) and, although not statistically significant, Peru had 64% greater likelihood of PrEP discontinuation (1.64 [95% CI, .98–2.74]; *P* = .06) (Table 4).

**Table 4.** Multivariate Model of Factors Associated With Preexposure Prophylaxis (PrEP) Discontinuation Among In-Study PrEP Initiators

Variable^a^	HR (95% CI)	*P* Value
Age, per 10 y	0.89 (.74–1.06)	.195
Gender identity		
Cisgender male	1.00 (ref.)	
Transgender male	0.58 (.08–4.31)	.597
Transgender female	1.69 (.87–3.28)	.125
Other/not assessed	1.59 (.84–3.01)	.153
Sexual orientation		
MSM	1.00 (ref.)	
Bisexual	1.29 (.91–1.83)	.149
Straight	1.21 (.36–4.04)	.756
Other/not assessed	1.63 (1.00–2.67)	.052
Race		
White	1.00 (ref.)	
Black or African American	1.18 (.85–1.65)	.316
Other	1.16 (.75–1.80)	.500
Combined^b^	0.96 (.61–1.52)	.866
Hispanic or Latino	1.34 (.95–1.90)	.096
Country		
United States	1.00 (ref.)	
Switzerland	1.17 (.43–3.18)	.765
Brazil	0.37 (.22–.60)	<.001
Peru	1.64 (.98–2.74)	.061

Abbreviations: CI, confidence interval; HR, hazard ratio; MSM, men who have sex with men.

^a^Variables considered for least absolute shrinkage and selection operator selection: treatment, age, male sex at birth, gender identity, sexual orientation, race, Hispanic or Latino, region, history of prior preexposure prophylaxis use, baseline behavioral risk score.

^b^Combined: racial categories that accounted for less than 5% of the study population. These include American Indian/Alaska Native, Asian, multiracial, and Native Hawaiian/Other Pacific Islander.

## DISCUSSION

Among AMP study participants who were mostly young MSM, about one-third initiated oral PrEP after study enrollment. Individuals with prior history of oral PrEP use, Brazil participants, and US participants enrolling at sites located in areas with higher unmet PrEP need had a higher likelihood of initiating oral PrEP. Notably, among all PrEP initiators, more than one-third discontinued PrEP at least once during study follow-up. Participants from Brazil had a lower likelihood of PrEP discontinuation, and there was a trend toward a higher likelihood of discontinuation among participants from Peru. Importantly, individual characteristics including age, race, ethnicity, sexual orientation, or ethnicity showed no association with PrEP initiation or PrEP discontinuation, suggesting that contextual factors may have a larger impact on PrEP initiation and discontinuation than individual factors.

As expected, providing oral PrEP access to AMP participants reduced HIV acquisition among PrEP initiators. Despite this, HIV acquisition endpoints were still met, likely related to heterogeneous regional PrEP initiation and discontinuation, and lower PrEP effectiveness than has been observed in oral PrEP efficacy clinical trials [2]. Overall, oral PrEP initiation, discontinuation, and effectiveness in our study mirrored published data in real-life settings. For example, in 2019, 23% of PrEP-eligible individuals were on PrEP in the US [17]. Likewise, our measured oral PrEP discontinuation over a 2-year period of 34.5% coincides with what was reported in a recent meta-analysis, where the overall proportion of oral PrEP discontinuation across 59 studies was 35% 1 year after PrEP initiation [18]. Last, in nonclinical trial settings, oral PrEP effectiveness ranges from 60% to 80% depending on adherence [19]. These important data show that offering oral PrEP as standard of prevention in future HIV-prevention, placebo-controlled efficacy trials will likely reduce HIV incidence among study participants who initiate and persist on PrEP, while also allowing for study endpoints to be met, given the ebbs and flows of oral PrEP utilization in real-life settings. Our data also emphasize the importance of PrEP uptake and adherence counseling among study participants in placebo-controlled trials, to optimize their safety during study participation.

As with the initial PrEP analysis for the AMP studies, we observed significant regional variations in PrEP initiation. Different regional levels of participants’ PrEP awareness and willingness to use PrEP may have contributed to the observed differences [20, 21]. The AMP studies were conducted between 2016 and 2019. At that time, PrEP awareness among MSM ranged between 80% and 90% in the US [22, 23], 79.8% in Switzerland [24], 69% in Brazil, and 46.6% in Peru, while willingness to use was reported at 60% in the US [23], 77.9% in Switzerland [24], 62% in Brazil, and 57.6% in Peru [20, 21]. Higher informational barriers related to side effects and PrEP effectiveness, as well as PrEP-related stigma, were observed among Peruvian MSM and TGW compared to their Brazilian counterparts [20]. The existence of a government-based initiative to implement PrEP at a national level may have also impacted PrEP initiation at different AMP regions, by way of increasing PrEP awareness and willingness to use. In 2017, based on the results of the PrEP Brasil Demonstration Project showing high PrEP uptake, adherence, and persistence [25], the Brazilian Ministry of Health approved oral PrEP to be offered to key populations at no cost as part of their national health system [26]. In Peru, PrEP has not been adopted as public policy by the Ministry of Health to date, and as a result, it is only available via demonstration projects or to those who can afford to purchase medication and pay for private care outside the public health system [27, 28]. In the US, higher unmet PrEP need, measured by the PrEP-to-need ratio corresponding to each site, was associated with higher PrEP initiation, suggesting that the AMP study reduced barriers impeding PrEP access in some areas of the US and resulted in increased uptake.

As in our study, regional differences in PrEP discontinuation were also observed in ImPrEP, an open-label, multicenter implementation study of same-day PrEP access in Brazil, Mexico, and Peru [29]. In ImPrEP, Peruvian participants had higher odds of early loss to PrEP follow-up and higher HIV incidence compared to Mexico and Brazil. Differences in the enrolled study populations may have impacted these outcomes. Compared to participants enrolled in Brazil, individuals enrolled in ImPrEP in Peru had a higher proportion of TGW (11% vs 5%) and greater involvement in transactional sex (22.4% vs 10%). TGW in Peru, as in most of Latin America, face significant social and structural barriers to PrEP uptake and long-term persistent use, including poverty, exclusion from the formal labor market, low educational attainment, systemic discrimination in the public health system related to their gender identity, and scarcity of gender-affirming care [30–32]. Peruvian enrollees also had a lower proportion of directly seeking PrEP at study entry compared to Brazilian enrollees (63.1% vs 96.1%), thought to be related to lower PrEP awareness in Peru compared to Brazil, especially among TGW [29, 32].

This study has several limitations. First, as PrEP-related data were obtained using the PrEP/PEP clinical research forms and were limited to what was reported by the clinical site staff, which did not include medication adherence information. Second, our study characterized oral PrEP initiation and its effect on HIV incidence. Our results are not applicable to newer long-acting injectable formulations of PrEP, where adherence to taking daily pills does not play a role in its efficacy. As such, it remains unknown if offering long-acting injectable PrEP would be a feasible option for future HIV prevention efficacy clinical trials given its superiority in reducing HIV incidence compared to daily oral PrEP as demonstrated in clinical trials [3]. Last, PrEP awareness, willingness to use, and the overall PrEP accessibility landscape in the US, Switzerland, and Latin America has evolved since the AMP study was launched, and as such, results of PrEP initiation, discontinuation, and their effects on clinical trial study outcomes may be different now.

Despite these limitations, this study offers a nuanced characterization of PrEP initiation and discontinuation among participants enrolled in a placebo-controlled HIV prevention efficacy clinical trial. Data from this study on patterns of PrEP initiation, discontinuation, and effects on HIV incidence outcomes can inform the design of future placebo-controlled studies. Our findings can help investigators who are developing new placebo-controlled HIV-prevention clinical trials to be more comfortable using local PrEP data to estimate HIV incidence and in-study oral PrEP uptake. For future trials planning to add oral PrEP as standard of prevention, site selection must take into consideration local PrEP utilization patterns and its associated factors, including community acceptability, local accessibility, and cost. Strategies to address these factors can then be developed during study design and implemented during study conduct. Last, to maintain ethical benchmarks of conducting clinical trials in low- and middle-income countries, investigators and sponsors need to advocate for equitable access of the tested products after the trial ends, if proven efficacious and safe [33].

## CONCLUSIONS

Regional PrEP accessibility conditions impacted oral PrEP initiation, discontinuation, and effectiveness, when included as standard of prevention in the HVTN 704/085 study. Future placebo-controlled HIV prevention efficacy clinical trials planning to add oral PrEP as standard of prevention should consider local PrEP utilization patterns and prioritize populations that are likely to benefit the most from future HIV prevention modalities.

## Supplementary Material

ofae387_Supplementary_Data

## Supplementary Data

Supplementary materials are available at *Open Forum Infectious Diseases* online. Consisting of data provided by the authors to benefit the reader, the posted materials are not copyedited and are the sole responsibility of the authors, so questions or comments should be addressed to the corresponding author.

## Notes

***Acknowledgments.*** The authors would like to acknowledge the HVTN Scientific Leadership Development Program, the AMP Protocol Team, the participants in HVTN 704/HPTN 085, AMP study Clinical Research Site staff including their Community Engagement and Recruitment teams without whom this study would not have been completed, and the clinical research sites’ Community Advisory Boards.

***Financial support*.** This analysis was supported by the National Institute of Allergy and Infectious Diseases through the following awards: UM1 AI068614, to the HVTN; UM1 AI068635, to the HVTN Statistical Data and Management Center (SDMC), Fred Hutchinson Cancer Research Center (FHCRC); UM1 AI068618, to HVTN Laboratory Center, FHCRC; UM1 AI068619, to the HPTN Leadership and Operations Center; UM1 AI068613, to the HPTN Laboratory Center; and UM1 AI068617, to the HPTN SDMC.
