# Supplementary material for: Oral Preexposure Prophylaxis Uptake and Discontinuation in the HIV Vaccine Trials Network 704/HIV Prevention Trials Network 085 Study: Implications for Biomedical Human Immunodeficiency Virus Prevention Trials
Source: Open Forum Infect Dis. 2024 Jul 16;11(7):ofae387. doi: 10.1093/ofid/ofae387 (PMC11259185; doi:10.1093/ofid/ofae387)
Supplement: ofae387_Supplementary_Data [file ofae387_supplementary_data.docx]

**SUPPLEMENTAL MATERIAL**

**Supplemental Table 1: PrEP Initiation and Discontinuation by region among AMP participants**

| Region | Not using PrEP at enrollment | N Initiated PrEP | % Initiated PrEP | N Discontinued PrEP | % Discontinued PrEP |  |
| --- | --- | --- | --- | --- | --- | --- |
| **US, Total** | 951 | 516 | 54.2% | 183 | 35.4% |  |
| US, Northeast | 465 | 240 | 51.6% | 92 | 38.3% |  |
| US, Midwest | 67 | 19 | 28.4% | 9 | 47.4% |  |
| US, South | 301 | 207 | 68.8% | 60 | 28.9% |  |
| US, West | 118 | 50 | 42.4% | 22 | 44.0% |  |
| **Switzerland** | 34 | 13 | 38.2% | 4 | 30.8% |  |
| **Brazil** | 143 | 119 | 83.2% | 22 | 18.5% |  |
| **Peru** | 1,093 | 58 | 5.3% | 34 | 58.6% |  |
| **TOTAL** | 2,221 | 706 | 31.8% | 243 | 34.4% |  |

**Supplemental Table 2:** **Multivariate Model of Factors Associated with PrEP Initiation (US sites only)**

| **Variable^*^** | HR (95% CI) | p |
| --- | --- | --- |
| **Male sex at birth** | 1.70 (0.74, 3.89) | 0.208 |
| **Sexual orientation** |  |  |
| Men who have sex with men | 1.00 (ref.) |  |
| Bisexual | 0.92 (0.72, 1.18) | 0.522 |
| Straight | 0.94 (0.42, 2.10) | 0.876 |
| Other/Not assessed | 0.70 (0.48, 1.00) | 0.052 |
| **Self-reported PrEP use and discontinuation prior to enrollment** | 1.95 (1.07, 3.54) | 0.029 |
| **PrEP to need ratio (US only), per 5 units** | 0.90 (0.84, 0.97) | 0.007 |

^*^ Variables considered for LASSO selection: treatment, age, male sex at birth, gender identity, sexual orientation, race, Hispanic or Latino/a, region, history of prior PrEP use, baseline behavioral risk score

**Supplemental Table 3: HIV incidence rates before and after PrEP initiation**

|  | Person-Years (PY) | Number of HIV diagnoses | Rate per 100 PY (95% CI) |
| --- | --- | --- | --- |
| Before PrEP initiation | 2,368 | 86 | 3.6 (2.9-4.5) |
| After PrEP initiation | 864 | 10 | 1.2 (0.6-2.1) |

**Supplemental Table 4: Days to study termination for PrEP initiators and non-initiators (censored at week 80)**

|  | Days | median (range) |
| --- | --- | --- |
| PrEP non-initiator | 1,515 | 562 (21 to 771) |
| PrEP initiator | 706 | 577 (28 to 801) |

**Supplemental Table 5: Multivariate model of the effect of PrEP initiation on study non-retention**

|  | HR (95% CI) | p |
| --- | --- | --- |
| **PrEP initiation** | 1.04 (0.78, 1.39) | 0.776 |
| **Age, per 10 years** | 0.74 (0.61, 0.90) | 0.002 |
| **Gender identity** |  |  |
| Cisgender male | 1.00 (ref.) |  |
| Transgender male | 1.91 (0.61, 6.01) | 0.267 |
| Transgender female | 1.52 (1.06, 2.18) | 0.022 |
| Other/Not assessed | 1.17 (0.64, 2.14) | 0.611 |
| **Hispanic or Latino/a** | 0.89 (0.60, 1.30) | 0.538 |
| **Country** |  |  |
| United States | 1.00 (ref.) |  |
| Switzerland | 0.41 (0.10, 1.65) | 0.207 |
| Brazil | 0.92 (0.52, 1.60) | 0.761 |
| Peru | 1.26 (0.78, 2.02) | 0.350 |
| **Baseline BRS*, per 1 unit** | 1.05 (0.87, 1.27) | 0.614 |

^*^BRS: Behavioral Risk Score. Higher numbers indicate higher likelihood of HIV acquisition.
